# Community-Onset Fungemias: Epidemiology and Genomic Characterization at a Tertiary-Care Hospital in Barcelona, Spain

**DOI:** 10.3390/jof11110808

**Published:** 2025-11-14

**Authors:** Celso Soares Pereira Batista, Alba Rivera, Maria Teresa Alvarez Albarran, Marc Rubio, Iris Belen-Figas, Cristina Lopez-Querol, Elisenda Miró, Ferran Navarro, Ferran Sanchez-Reus

**Affiliations:** 1 Department of Genetics and Microbiology, Autonomous University of Barcelona, 08193 Bellaterra (Cerdanyola del Vallès), Spain; mrivera@santpau.cat (A.R.); emiro@santpau.cat (E.M.); fnavarror@santpau.cat (F.N.); 2 Microbiology Department, Hospital de la Santa Creu i Sant Pau, 08041 Barcelona, Spain; mrubiobu@santpau.cat (M.R.); clopezq@santpau.cat (C.L.-Q.); fsanchezr@santpau.cat (F.S.-R.); 3 Sant Pau Biomedical Research Institute (IIB Sant Pau), 08041 Barcelona, Spain; irisbelen20009@gmail.com; 4 Emergency Department, Hospital de la Santa Creu i Sant Pau, 08041 Barcelona, Spain; malvareza@santpau.cat

**Keywords:** community-onset fungemia, candidemia, *Candida* species, antifungal resistance, whole-genome sequencing

## Abstract

**Background**: Community-onset fungemia is a clinically significant syndrome frequently linked to recent healthcare exposure and significant morbidity and mortality. Methods: We performed a 21-year, single-centre retrospective cohort of consecutive yeast bloodstream infections diagnosed at the Emergency Department (2004–2024). Clinical/epidemiological data, species identification (MALDI-TOF MS), antifungal susceptibility (CLSI M27; Sensititre YO10), and whole-genome sequencing (WGS) were analyzed. **Results**: Forty-eight episodes (51 isolates) were included; 56.3% were male, median age 74 years (IQR 63–82). Acquisition was healthcare-associated in 38/48 (79.2%). Sources were unknown (36.7%), abdominal (22.4%), urological (22.4%), catheter-related (14.3%), and 2.1% was attributed to a cardiovascular and a joint focus; 18.8% were polymicrobial. Crude mortality was 20.8% at 7 days (10/48) and 29.2% at 30 days (14/48). Species distribution: *Candida albicans* 41.2%, *Nakaseomyces glabratus* 27.5%, *Candida parapsilosis* 11.8%, *Candida tropicalis* 11.8%, *Pichia kudriavzevii* 3.9%, *Clavispora lusitaniae* 1.9%, and *Candida orthopsilosis* 1.9%. No isolate was resistant to anidulafungin, micafungin, or amphotericin B; one *N. glabratus* showed reduced susceptibility to caspofungin. Azole resistance was observed in one *C. albicans* and one *N. glabratus* isolate. WGS (44 isolates) confirmed MALDI-TOF identifications and characterized resistance markers. All 12 sequenced *N. glabratus* carried ERG2 I207V, PDR15/PDH1 E839D, and PDR1 V91I/L98S. Notable cases included one *N. glabratus* caspofungin-intermediate with FKS2 F659C, *N. glabratus* fluconazole-resistant with multiple PDR1 substitutions including a unique novel G857V, and *C. albicans* fluconazole-resistant harbouring alterations in MRR1/MRR2, CDR1, and ERG11. **Conclusions**: In this 21-year cohort, community-onset fungemia was predominantly healthcare-associated, with *C. albicans* as the predominant species, followed by *N. glabratus*. Crude mortality reached 29.2% at 30 days. Echinocandin resistance was not observed; azole resistance was uncommon. WGS provided precise speciation and actionable insight into resistance mechanisms, including a putatively novel PDR1 G857V in *N. glabratus*.

## 1. Introduction

Community-onset fungemia, characterized by a positive fungal blood culture obtained within 48 h of hospital admission, is an important clinical condition linked with significant morbidity and mortality [1,2].

According to some studies, community-onset cases represent approximately one-third of all candidemia episodes [2,3]. Patients with community-onset fungemia frequently have recent healthcare exposure, with studies reporting that up to 75% were hospitalized in the prior three months [2]. Delayed antifungal therapy (median 2.7 days) contributes to a high 30-day mortality (~26%) [2]. By contrast, classic nosocomial candidemia (arising ≥ 48 h into admission) typically involves the ICU and invasive devices [4,5]. Distinguishing community-onset cases highlights the need to recognize at-risk patients early.

Diagnostic delays remain a significant limitation. Blood cultures, currently considered the standard method, are slow and fail to detect a substantial proportion of cases, with positivity rates reported between 21% and 71% in proven invasive candidiasis [6]. Even when cultures yield growth, several additional days are usually required for species identification and antifungal susceptibility testing, further postponing timely optimization of therapy.

*Candida* is the leading genus responsible for fungal bloodstream infections [7,8,9]. When these infections are caused by *Candida* spp., they are defined as candidemia [10,11].

The distribution of *Candida* species responsible for candidemia varies across geographic regions and hospital settings. These differences reflect not only institutional factors but also patient-related conditions, such as underlying diseases, prior antifungal exposure, and other risk factors that shape local epidemiology. According to recent surveillance data, five species (*C. albicans*, *C. glabrata*, *C. tropicalis*, *C. parapsilosis*, and *C. krusei*) account for the vast majority of candidemia cases, collectively representing more than 90% of isolates [12,13,14,15].

Recent taxonomic revisions, enabled by advances in molecular biology, have reclassified several clinical *Candida* species into new genera. For example, *Candida glabrata* is now *Nakaseomyces glabratus*, *C. krusei* is *Pichia kudriavzevii*, and *C. lusitaniae* is *Clavispora lusitaniae*. These changes reflect the fact that the former genus *Candida* included many morphologically similar but genetically unrelated yeasts.

Historically, species were grouped together based on shared phenotypic traits such as budding cells, white colonies, and the absence of a known sexual stage. However, molecular analyses have revealed that these yeasts belong to several distinct, well-defined evolutionary lineages that better fit modern taxonomic principles. Consequently, the term “candidemia” now encompasses species that no longer formally belong to the *Candida* genus, creating some ambiguity in clinical communication and laboratory reporting. This rapid pace of taxonomic change, particularly among ascomycetous yeasts, has prompted active discussion within the medical mycology community regarding its impact on clinical practice and diagnostic workflows [16].

In terms of susceptibility, community-onset isolates generally reflect the species profile, with most remaining susceptible to first-line antifungals, although non-*albicans* species often show reduced susceptibility to azoles. Echinocandin resistance remains uncommon in both hospital- and community-acquired isolates. Whole-genome sequencing (WGS) is reshaping the capacity to detect antifungal resistance and accurately delineate fungal species, enabling the precise identification of cryptic species together with the simultaneous detection of resistance mutations. Compared with traditional methods, WGS provides faster and more scalable analysis and has contributed to uncovering both known and novel genetic determinants of resistance [17].

Our study, therefore, aims to describe the epidemiological, clinical, and molecular characteristics of yeast bloodstream infections diagnosed over 21 years at a tertiary hospital. WGS was applied to provide precise species identification and to characterize antifungal resistance mechanisms.

## 2. Materials and Methods

### 2.1. Clinical and Epidemiological Data

A retrospective cohort study was conducted at Hospital de la Santa Creu i Sant Pau, a tertiary and university hospital in Barcelona, Spain, with 548 beds, serving a reference population of approximately 407,000 inhabitants and receiving around 160,000 Emergency Department visits per year, encompassing all patients diagnosed with fungemia (restricted to pathogenic yeasts including *Candida* species and reclassified genera such as *Nakaseomyces*, *Clavispora*, and *Pichia*, while excluding *Cryptococcus* spp.).

Community-onset fungemia was defined as a bloodstream infection diagnosed within the first 48 h of hospital admission. These cases were further classified as healthcare-associated (recent hospitalization, surgery, dialysis, or residence in a care facility) or community-acquired (no recent healthcare contact).

Relevant clinical and epidemiological data, including patient demographics, underlying conditions, and outcomes, were collected from medical records. Cases were identified via the hospital’s microbiology and admissions databases, and data were compiled for analysis with appropriate confidentiality safeguards. The study received approval from the hospital’s Ethics Committee (IIBSP-WIS-2022-144; approved on 17 April 2024).

Clinical variables were defined as follows (Table 1):

### 2.2. Identification and Antifungal Susceptibility

Viable yeast isolates obtained from blood cultures were preserved by lyophilization, with four vials per isolate stored for long-term maintenance. For initial recovery, a lyophilized vial of each isolate was rehydrated in sterile saline and streaked onto ChromAgar Candida plates (CHROMagar™, Paris, France), followed by incubation at 35–37 °C for 48 h. This chromogenic medium supports robust yeast growth and produces distinctive colony colours for presumptive identification of different Candida species after 48 h. If an isolate failed to grow after two attempts on ChromAgar, an alternative recovery method was employed: the lyophilized vial was rehydrated in sterile saline and then inoculated into an aerobic blood culture bottle (BacT/ALERT VIRTUO system, bioMérieux, Marcy-l’Étoile, France) and incubated until flagged as positive. Once the blood culture bottle was flagged as positive, its contents were examined by Gram staining to verify the presence of yeast cells. Subsequently, an aliquot was subcultured onto ChromAgar Candida to recover and isolate yeast colonies. All recovered yeast isolates, whether obtained via direct plating or blood culture enrichment, were maintained with serial subcultures on ChromAgar Candida to ensure purity and viability. Subcultures were performed every 48–72 h (two sequential passages) prior to identification and susceptibility testing. Species-level identification of the yeasts was achieved by matrix-assisted laser desorption/ionization time-of-flight mass spectrometry (MALDI-TOF MS) using MALDI Biotyper^®^ sirius GP System (Bruker Daltonik GmbH, Bremen, Germany). Antifungal susceptibility testing was carried out by broth microdilution using the Sensititre YeastOne YO10 colorimetric panel (Thermo Fisher Scientific, Waltham, MA, USA); endpoints were interpreted according to Clinical and Laboratory Standards Institute (CLSI) species-specific breakpoints (M27 guidelines [18]). For *Candida orthopsilosis* (a member of the *C. parapsilosis* species complex lacking its own breakpoints), the CLSI recommends applying the breakpoints established for *C. parapsilosis*.

### 2.3. Molecular Analysis by Whole-Genome Sequencing (WGS)

#### 2.3.1. DNA Extraction

Genomic DNA was extracted from recovered isolates to enable molecular analyses. Extractions were performed using the DNeasy UltraClean Microbial Kit (Qiagen, Hilden, Germany) following the manufacturer’s protocol. The concentration of each DNA prep was assessed with a Qubit 4 fluorometer (Invitrogen, Thermo Fisher Scientific).

#### 2.3.2. Next-Generation Sequencing (NGS)

Purified DNA extracts were sent to Novogene Europe (Germany) for next-generation sequencing using an Illumina NocaSeq 6000 sequencer. Paired-end reads were generated and delivered as FASTQ files for downstream analysis.

#### 2.3.3. Bioinformatic Analysis

Raw paired-end reads were quality trimmed using fastp [19] and quality checked using FastQC (available at https://www.bioinformatics.babraham.ac.uk/projects/fastqc/, accessed on 30 May 2025) and MultiQC [20]. Trimmed reads were then assembled into contigs using SKESA [21] wrapped in shovill (available at https://github.com/tseemann/shovill, accessed on 6 June 2025).

Taxonomic identification was performed using GAMBIT [22]. To ensure correct taxonomic classification, 28S and ITS sequences were extracted from FASTA files using BLASTn (v2.16.0+) [23], and taxonomic identification was then performed against the NCBI RefSeq Targeted Loci Project rRNA/ITS databases via the online BLASTn suite.

We used ChroQueTas [24], an open-source tool for rapid screening of fungal genomes for antimicrobial resistance (AMR) mutations, with the FungAMR database.

The combined use of automated tools and manual curation ensured comprehensive identification of both known and novel resistance mutations.

### 2.4. Data Analysis

Clinical and laboratory data were analyzed using R (v4.4.3) in RStudio. Descriptive statistics summarized patient characteristics. Categorical data were shown as counts and percentages; continuous data as medians with IQRs. Visualizations were produced with ggplot2.

## 3. Results

### 3.1. Epidemiology and Clinical

Annual incidence ranged from one to five cases without clear trends. A total of 48 cases were included in the study. The median age of patients was 74 years (interquartile range [IQR]: 63–82 years), and 56.3% were male (Table 2).

Prevalent clinical risk factors included recent antibiotic use within the previous 30 days (21 cases, 43.8%), presence of a central venous catheter (11 cases, 22.9%), history of solid tumours (15 cases, 31.2%), diabetes mellitus (15 cases, 31.2%), and recent abdominal surgery (9 cases, 18.8%). Additional factors included chemotherapy (10 cases, 20.8%), chronic kidney disease (eight cases, 16.7%), corticosteroid treatment (eight cases, 16.7%), hematological cancer (four cases, 8.3%), neutropenia (three cases, 6.3%), recent immunosuppressive therapy (three cases, 6.3%), recent hematopoietic transplant (two cases, 4.2%), persistent fungemia (four cases, 8.3%), and antifungal treatment within 30 days (two cases, 4.2%). These factors are not mutually exclusive, and some patients had none of the listed conditions.

Regarding the setting of acquisition, 10 cases (20.8%) were strictly community-acquired and 38 (79.2%) were healthcare-associated. The most frequent sources of fungemia included unknown origin (18 cases, 37.5%), urological infections (11 cases, 22.9), abdominal infections (10 cases, 20.8%), and catheter-related bloodstream infections (seven cases, 14.6%). Additionally, one case each was attributed to a cardiovascular and a joint focus.

Empirical antifungal therapy was administered in only one case (2.1%), receiving antibiotics and fluconazole. Empirical antibacterial therapy alone was prescribed in 40 cases (83.3%), while seven patients (14.6%) received no empirical treatment. Mortality was 20.8% (10 cases) at 7 days post-diagnosis and 29.2% (14 cases) at 30 days.

Monomicrobial fungemia was present in 39 cases (81.2%), while nine cases (18.8%) were polymicrobial: seven involved concurrent bacteremia and two included multiple *Candida* species. Among these, one case involved two different *Candida* spp. (*C. albicans* and *C. parapsilosis*) species and another three distinct yeasts (*C. albicans*, *C. tropicalis* and *N. glabratus*). In total, 51 isolates were recovered from the 48 patients.

### 3.2. Identification and Antifungal Susceptibility Test

The most frequently isolated species was *C. albicans* (21 isolates, 41.2%), followed by *N. glabratus* (14 isolates, 27.5%), *C. parapsilosis* (six isolates, 11.8%), *C. tropicalis* (six isolates, 11.8%), and *P. kudriavzevii* (two isolates, 3.9%). Single isolates were identified as *C. lusitaniae* and *C. orthopsilosis* (1.9% each).

**Figure 1:**
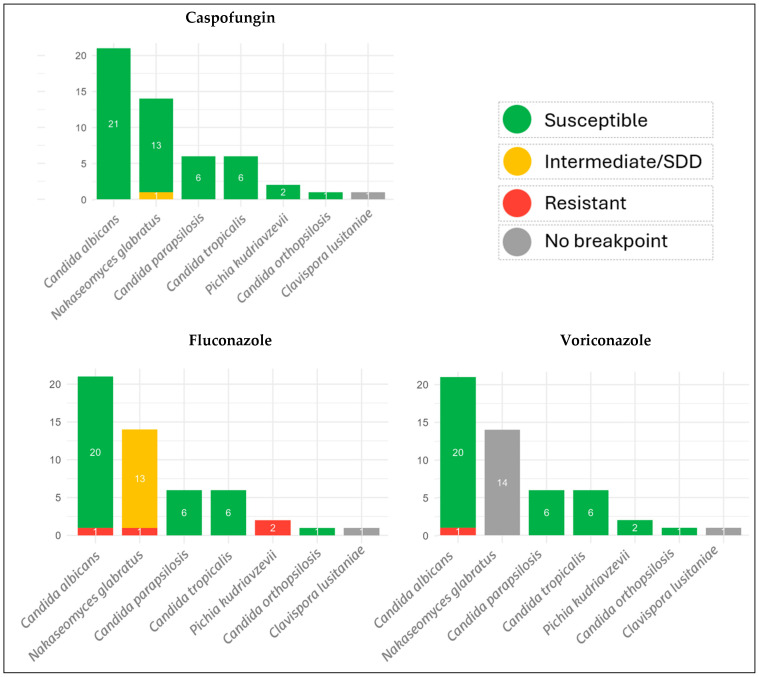
Antifungal susceptibility profiles of clinical isolates according to species.

None of the isolates showed resistance to anidulafungin, micafungin, or amphotericin B. Two *N. glabratus* isolates had reduced susceptibility to caspofungin (MIC = 0.25 μg/mL). Azole resistance was detected in two cases: one *C. albicans* isolate (voriconazole MIC > 16 μg/mL; fluconazole MIC > 256 μg/mL) and one *N. glabratus* isolate (voriconazole MIC > 16 μg/mL; fluconazole MIC = 128 μg/mL). *P. kudriavzevii* is intrinsically resistant to fluconazole (Figure 1).

### 3.3. WGS

Of the 51 isolates recovered, 45 were successfully sequenced. Among them, 19 corresponded to *C. albicans* (17 with mutations), 13 to *N. glabratus* (all with mutations), four to *C. parapsilosis* (one mutated), five to *C. tropicalis* (one mutated), two to *P. kudriavzevii* (without mutations), and the single isolates of *C. lusitaniae* and *C. orthopsilosis* (both without mutations). WGS could not be performed on the remaining six isolates due to insufficient DNA quantity or quality. No discrepancies were observed between MALDI-TOF identification and WGS results in the sequenced isolates.

The 13 sequenced *N. glabratus* isolates carried the mutations I207V in ERG2, E839D in PDR15, and V91I and L98S in PDR1. Table 4 summarizes all mutations previously described for *N. glabratus* identified in our study. Of the 18 previously reported mutations in *N. glabratus*, all were detected in susceptible strains except for the F659C substitution in FKS2, which was present only in the caspofungin-intermediate strain. The detailed summary of resistance-related genes analyzed and key mutations identified by WGS is provided in the Supplementary Table S1. Table 3 provides a summary of all identified mutations that had been previously reported for *N. glabratus*.

Of the 18 previously reported mutations in *N. glabratus*, all were detected in susceptible strains except F659C, which was found only in the caspofungin-intermediate strain. Two *N. glabratus* isolates were of clinical interest due to their resistance phenotypes. One caspofungin-intermediate isolate carried mutations in the glucan synthase genes FKS2 as well as FKS3 (R1039L, N1825S). The other, one fluconazole-resistant isolate, showed alterations in efflux pump regulators (PDR1 S76P, V91I, L98S, T143P, G857V; CDR1 H58Y; PDH1/PDR15 E839D) and in ergosterol pathway genes (ERG2 I207V, ERG8 N448S). The only mutation unique to this isolate was the substitution G857V in PDR1. Table 4 summarizes the novel mutations identified in *N. glabratus*.

In the case of *C. albicans*, 16 previously reported mutations were detected, all of which were neutral. Similarly, in *C. parapsilosis*, another neutral mutation was identified in ERG11 (R398I) in two fluconazole-susceptible isolates. Table 5.

The only *C. albicans* fluconazole-resistant strain showed multiple mutations affecting transcriptional regulators CDR1, MRR2 and the ergosterol biosynthetic enzyme ERG11. Some of these mutations appeared to be unique to this strain (Table 6).

## 4. Discussion

Our study provides an integrated clinical, epidemiological, and genomic analysis of community-onset candidemia cases diagnosed over a 21-year period at a single tertiary-care hospital. Despite the long study period, no temporal trend was observed, likely due to the low annual case numbers and the sporadic nature of community-onset candidemia. Only a limited number of studies have examined community-onset candidemia [1,2]. These infections are more frequently nosocomial in origin, and most cases that appear to originate in the community are in fact linked to recent healthcare exposure. Our data are consistent with those reported in the literature, with most cases being healthcare-associated [2].

The clinical profile of our cohort reflects the risk factors most frequently described in the literature for candidemia [35], including recent antibiotic exposure [36,37], central venous catheters [38,39], malignancy [40], and recent surgery [41]. However, in our series, no single factor accounted for more than one-third of cases, making clinical suspicion difficult and often hindering the timely initiation of appropriate empirical antifungal therapy.

Several clinical prediction scores have been proposed to estimate the risk of candidemia in hospitalized patients, including the *Candida* Score [42] and the Ostrosky-Zeichner rule [43]. These tools incorporate factors such as severe sepsis, recent surgery, central venous catheter, total parenteral nutrition, and broad-spectrum antibiotic use. They show a high negative predictive value, making them useful for ruling out infection, although their positive predictive value is low [44,45]. While they have been primarily validated in ICU populations, their components overlap with the risk factors observed in our cohort, suggesting that they could aid in the early identification of patients at higher risk of candidemia. However, no score has yet been specifically validated for community-onset cases.

All-cause mortality in our series was high, at 20.8% at 7 days and 29.2% at 30 days after diagnosis. These figures are consistent with previous reports in community-onset candidemia cohorts and reflect the severity of this condition, although it remains infrequent [46,47,48].

Species distribution varies depending on age, underlying disease, or geographic region [35,49,50], with *C. albicans* being the most frequent species in Europe. In our cohort, after *C. albicans*, the second most frequent species was *N. glabratus* and *C. parapsilosis*, consistent with a recent European multicentre study [51].

Echinocandins are the recommended first-line therapy for candidemia, given their fungicidal activity and favourable safety profile [11,52]. In our study, we did not observe echinocandin resistance, aligning with contemporary surveillance showing low echinocandin resistance [53,54].

The low proportion of resistant strains observed in our cohort may be explained by the limited antifungal pressure in the community setting, the relatively infrequent prior exposure to antifungal agents among patients, and the overall low antifungal resistance rates reported in our institution.

Echinocandin resistance in *N. glabratus* arises from amino-acid substitutions in conserved hotspot regions of FKS1 and especially FKS2, which encode subunits of the 1,3-β-D-glucan synthase complex. One caspofungin-intermediate *N. glabratus* harboured the hotspot substitution FKS2 F659C, which is well established to confer reduced susceptibility to echinocandins [27,55,56]. By contrast, FKS3 is expressed at least 100-fold lower than FKS1, is non-essential for vegetative growth, and its deletion does not change echinocandin MICs, indicating no established role in clinical echinocandin resistance [57].

In *N. glabratus*, azole resistance is most commonly mediated by gain-of-function mutations in the transcription factor PDR1, which upregulates the expression of ATP-binding cassette (ABC) efflux pump genes such as CDR1, PDH1 (CDR2), and SNQ2 [58,59,60]. These mutations promote overexpression of efflux pumps, leading to reduced intracellular azole accumulation and cross-resistance.

Our fluconazole-resistant *N. glabratus* isolate carried previously reported substitutions in PDR1 (S76P, V91I, L98S, T143P) [56], as well as CDR1 (H58Y) [23,54,55] and PDH1/PDR15 (E839D) [23,54], all of which are considered polymorphisms not known to confer azole resistance. The only mutation unique to this isolate was PDR1 G857V, which has not been previously reported and whose functional impact remains unknown.

Although mutations in ergosterol biosynthesis genes such as ERG2 or ERG8 have occasionally been reported to affect azole susceptibility, their role in resistance is less well defined [51,57]. *N. glabratus* can adapt to azole stress by altering sterol composition, including uptake of exogenous sterols when ergosterol biosynthesis is impaired, which may limit the impact of such mutations. Our isolate also harboured polymorphisms in ERG2 (I207V) [23] and ERG8 (N448S) [56], which are known not to confer azole resistance.

In *C. albicans*, azole resistance is commonly mediated by gain-of-function mutations in the transcription factor MRR1, which activates MDR1 expression, leading to efflux-mediated resistance. Additional regulators such as MRR2 can modulate CDR1 expression and contribute to azole resistance [28,31]. Our fluconazole-resistant *C. albicans* isolate harboured multiple mutations in efflux regulators (MRR1: E1020Q, V27del, PQS166 insertion, L248V, E336del, V340E; MRR2: L144V, T145A, S165N, S480P, I204del, S580del), the target enzyme ERG11 (Q142del), and the efflux pump CDR1 (T365del, Q790del, T947S, E949P, N1499stop, K1500del, K1501del). However, most CDR1 changes, including C-terminal deletions, have been reported across isolates with varying azole susceptibility and are not considered resistance-associated. Similarly, ERG11 Q142del lies outside recognized hotspots (e.g., Y132/K143) and alone does not predict resistance [30,61].

Taken together, the combination of MRR1/MRR2 alterations with the ERG11 indel (Q142del) provides a plausible genetic basis for the fluconazole-resistant phenotype in CAC_937, whereas the functional contribution of the individual CDR1 changes (including N1499stop/K1500–K1501del) remains uncertain and would require targeted experimental validation.

The study’s strengths lie in its two-decade surveillance, integration of clinical and genomic data, and identification of novel resistance markers. However, limitations include its retrospective, single-centre design, modest sample size, and lack of functional validation of mutations. Despite these constraints, the findings underscore the value of WGS in resistance surveillance and the ongoing clinical burden of community-onset fungemia, supporting the need for multicentre studies to confirm and expand these results.

This 21-year single-centre cohort provides an integrated clinical, epidemiological, and genomic view of community-onset fungemia. Most episodes were healthcare-associated, with *C. albicans* as the leading species, followed by *N. glabratus*. Mortality remained high (29.2% at 30 days). While echinocandin resistance was absent and azole resistance uncommon, WGS confirmed species identification, revealed both known and novel resistance-associated mutations.

## Figures and Tables

**Table 1:** Definition of clinical variables.

Variable	Definition	Value
Diabetes mellitus	Documented history of diabetes	Yes/No
Chronic kidney disease	Established diagnosis of chronic renal disease	Yes/No
Recent surgery	Surgical procedure within the previous three months; if yes, specify localization: abdominal, neurosurgical, or joint…	Yes/No
Hematological cancer	History of hematological malignancy	Yes/No
Neutropenia	Absolute neutrophil count below 500 cells/µL	Yes/No
Hematological transplantation	Prior hematopoietic stem-cell transplant	Yes/No
Solid tumour	Diagnosis of solid-organ malignancy, such as carcinomas, sarcomas, or other non-hematological cancers	Yes/No
Corticosteroid therapy	Administration of corticosteroids within 30 days prior to diagnosis	Yes/No
Chemotherapy	Receipt of chemotherapy within 30 days prior to diagnosis	Yes/No
Other immunosuppressive therapy	Use of non-steroidal, non-chemotherapeutic immunosuppressive agents such as calcineurin inhibitors, antimetabolites, mTOR inhibitors, Janus kinase inhibitors, or monoclonal antibodies such as anti-TNF or anti-CD20 within 30 days prior to diagnosis	Yes/No
Recent antibiotic use	Administration of antibiotics within 30 days prior to diagnosis	Yes/No
Recent antifungal use	Administration of antifungal agents within 30 days prior to diagnosis	Yes/No
Central venous catheter	Presence of a central venous catheter at the time of diagnosis	Yes/No
Urinary catheter	Presence of an indwelling urinary catheter, such as a Foley or bladder catheter/vesical catheter, at the time of diagnosis	Yes/No
Haemodialysis catheter	Presence of a vascular catheter specifically for haemodialysis	Yes/No
Healthcare-associated fungemia	Diagnosis within 48 h of admission in patients with recent healthcare contact: hospitalization within 3 months, surgery, dialysis, or residence in a long-term care facility	Yes/No
Community-acquired fungemia	Diagnosis within 48 h of admission in patients without recent healthcare exposure	Yes/No
Source of fungemia	First assessed clinically; considered positive if the same microorganism is isolated at the suspected focus. Sources are classified as “unknown” when neither compatible clinical features nor microbiological confirmation at the focus are present. Possible sources include unknown, urological, abdominal, catheter-related, cardiovascular, joint, gynecological, or cutaneous	Multinomial
Mixed Fungemia	Presence of more than one microorganism isolated in the same blood culture; if yes, classified as either multiple yeasts or yeast plus bacteria	Yes/No
Empirical treatment	Whether the patient received antimicrobial therapy before microbiological evidence of fungemia; if yes, specify if only antibiotic, only antifungal, or both in combination	Yes/No
Persistent fungemia	Positive blood cultures for yeast despite appropriate antifungal therapy	Yes/No
Recent antifungal use	Administration of antifungal agents within 30 days prior to diagnosis	Yes/No
Mortality at 7 days	Death occurring within 7 days of the fungemia diagnosis	Yes/No
Mortality at 30 days	Death occurring within 30 days of the fungemia diagnosis	Yes/No

**Table 2:** Demographics, epidemiological data, and clinical characteristics of the patients.

Characteristic	*n* = 48	Percent (%)
Median age in years ((interquartile range)	74 (63–82)	
Sex		
Female	21	43.7
Male	27	56.3
Predisposition factors		
Diabetes mellitus	15	31.3
Chronic kidney disease	8	16.7
Recent surgery (≤3 months)	11	22.9
Abdominal surgery	9	18.8
Neurosurgery	1	2.1
Joint surgery	1	2.1
Haematologic cancer	4	8.3
Neutropenia	3	6.3
Hematologic cell transplantation	2	4.2
Solid tumour	15	31.3
Corticosteroid therapy	8	16.7
Chemotherapy	10	20.8
Other immunosuppressive therapy	3	6.3
Recent antibiotic use	21	43.8
Recent antifungal use	2	4.2
Catheter presence	16	33.3
Central venous catheter	11	22.9
Urinary catheter	5	10.4
Haemodialysis catheter	1	2.1
Origin		
Community-acquired	10	20.8
Healthcare-associated	38	79.2
Source of fungemia		
Unknown	18	37.5
Urological	11	22.9
Abdominal	10	20.8
Catheter-related	7	14.6
Cardiovascular	1	2.1
Joint	1	2.1
Mixed Fungemia		
No	39	81.3
Mixed fungemia with bacteria	7	14.6
Mixed fungemia with different *Candida*	2	4.2
Empirical treatment		
Only antibiotic	40	83.3
Both antibiotic and antifungal	1	2.1
None	7	14.6
Persistent fungemia	4	8.3
Mortality		
Crude mortality at 7 days	10	20.8
Crude mortality at 30 days	14	29.2

**Table 3:** Summary of previously reported mutations in *N. glabratus* identified by WGS.

Function	Gene	Mutation	Isolates (n)	Resistant (n)	Previously Reported	Resistance Association	Ref.
Efflux Pump	CDR1	H58Y	4	F[R]	Yes	No	[25]
	FLR1(Ben1)	V254I	1	0	Yes	No	[25]
	PDH1(Pdr15)	T1530K	1	0	Yes	No	[25]
	PDH1(Pdr15)	E839D	12	F[R], C[I]	Yes	No	[25]
	PDR1	S76P	7	F[R], C[I]	Yes	No	[26]
	PDR1	D243N	5	0	Yes	No	[26]
	PDR1	V91I	12	F[R], C[I]	Yes	No	[25]
	PDR1	T143P	7	F[R], C[I]	Yes	No	[25]
	PDR1	L98S	12	F[R], C[I]	Yes	No	[25]
Ergosterol Pathway	ERG2	I207V	12	F[R], C[I]	Yes	No	[25]
	ERG4	T13N	1	0	Yes	No	[25]
	ERG6	R48K	1	0	Yes	No	[25]
	ERG7	T732A	2	C[I]	Yes	No	[25]
	ERG8	N448S	10	F[R], C[I]	Yes	No	[25]
Glucan Synthase	FKS2	F659C	1	C[I]	Yes	Yes	[27]
	FKS3	R1472Q	3	F[R]	Yes	No	[25]
Other	MSH2	V239L	3	0	Yes	No	[25]
	FEN1	M155T	2	0	Yes	No	[25]

Abbreviations: F[R] indicates one *N. glabratus* fluconazole-resistant isolate; C[I] indicates one *N. glabratus* caspofungin-intermediate isolate; MSH2: mismatch repair protein involved in DNA repair; FEN1: flap endonuclease 1, involved in DNA replication and repair.

**Table 4:** Summary of mutations not previously reported in the literature, identified in *N. glabratus*, identified by WGS.

Function	Gene	Mutation	Isolates (n)	Resistant (n)	Previously Reported	Resistance Association
Glucan Synthase	FKS3	R1039L	1	C[I]	No	Uncertain
	FKS3	N1825S	1	C[I]	No	Uncertain
	FKS3	A1621T	1	0	No	NM
Efflux Pump	PDR1	G857V	1	F[R]	No	Uncertain

Abbreviations: F[R] indicates one *N. glabratus* fluconazole-resistant isolate; C[I] indicates one *N. glabratus* caspofungin-intermediate isolate. NM: Neutral mutations were present in both resistant and susceptible isolates. Uncertain: The mutation was found only in a resistant isolate in our dataset, but its role in antifungal resistance is unclear based on currently available literature or functional data.

**Table 5:** Summary of previously reported mutations in *C. albicans* identified by WGS.

Function	Gene	Mutation	Isolates (n)	Resistant (n)	Previously Reported	Resistance Association	Ref.
Ergosterol Pathway	ERG11 CYP51	E266D	4	0	Yes	No	[28]
	ERG11 CYP51	V488I	1	0	Yes	No	[28]
	ERG11 CYP51	D116E	1	0	Yes	No	[28]
Transcription Factor	MRR2	S466L	1	0	Yes	No	[29]
	MRR2	T145A	8	F[R]	Yes	No	[30]
	MRR2	A468G	1	0	Yes	No	[30]
	MRR2	S480P	10	F[R]	Yes	No	[31]
	UPC2	I142S	9	0	Yes	No	[31]
	MRR2	S165N	9	F[R]	Yes	No	[28]
	MRR1	A880E	2	0	Yes	No	[30]
	MRR1	E1020Q	4	F[R]	Yes	No	[32]
	MRR2	V451A	4	0	Yes	No	[31]
	TAC1	M677del	6	0	Yes	No	[28]
	MRR1	L248V	1	F[R]	Yes	No	[33]
	MRR2	L144V	8	F[R]	Yes	No	[33]
	MRR1	NPQS166	1	F[R]	Yes	No	[34]

Abbreviations: F[R] indicates one *C. albicans* fluconazole-resistant isolate.

**Table 6:** Summary of mutations not previously reported in the literature, identified in *C. albicans* identified by WGS.

Function	Gene	Mutation	Isolates (n)	Resistant (n)	Previously Reported	Resistance Association
Efflux Pump	CDR1	V616F	3	0	No	NM
	CDR1	T673A	2	0	No	NM
	CDR1	A753K	1	0	No	NM
	CDR1	S539R	3	0	No	NM
	CDR1	T365del	1	F[R]	No	Uncertain
	CDR1	Q790del	1	F[R]	No	Uncertain
	CDR1	T947S	1	F[R]	No	Uncertain
	CDR1	E949P	1	F[R]	No	Uncertain
	CDR1	N1499	1	F[R]	No	Uncertain
	CDR1	K1500del	1	F[R]	No	Uncertain
	CDR1	K1501del	1	F[R]	No	Uncertain
Ergosterol Pathway	ERG11(CYP51)	Q142del	1	F[R]	No	Uncertain
Transcription Factor	CAP1	Q188dup	1	F[R]	No	Uncertain
	MRR1	V340E	1	F[R]	No	Uncertain
	MRR1	V27del	1	F[R]	No	Uncertain
	MRR1	E336del	1	F[R]	No	Uncertain
	MRR2	I204del	1	F[R]	No	Uncertain
	MRR2	S580del	1	F[R]	No	Uncertain

Abbreviation: F[R] indicates one *C. albicans* fluconazole-resistant isolate; NM: Neutral mutations were present in both resistant and susceptible isolates. Uncertain: The mutation was found only in a resistant isolate in our dataset, but its role in antifungal resistance is unclear based on currently available literature or functional data.

## Data Availability

De-identified clinical and laboratory datasets supporting the findings of this study are available from the corresponding author upon reasonable request and completion of a data-sharing agreement. WGS outputs (FASTQ/assemblies and key variant annotations) will be deposited in [NCBI SRA/ENA, BioProject ID to be added upon acceptance]; until then, they are available upon request.

## Supplementary Materials

The following supporting information can be downloaded at: https://www.mdpi.com/article/10.3390/jof11110808/s1, Table S1: Summary of clinical, antifungal, and WGS data.
